# Contemporary enterovirus-D68 isolates infect human spinal cord organoids

**DOI:** 10.1128/mbio.01058-23

**Published:** 2023-08-03

**Authors:** Gabrielle Aguglia, Carolyn B. Coyne, Terence S. Dermody, John V. Williams, Megan Culler Freeman

**Affiliations:** 1 Department of Pediatrics, Division of Infectious Diseases, University of Pittsburgh School of Medicine, Pittsburgh, Pennsylvania, USA; 2 Department of Molecular Genetics and Microbiology, Duke University School of Medicine, Durham, North Carolina, USA; 3 Institute for Infection, Inflammation, and Immunity (i4Kids), UPMC Children’s Hospital of Pittsburgh, Pittsburgh, Pennsylvania, USA; 4 Department of Microbiology and Molecular Genetics, University of Pittsburgh School of Medicine, Pittsburgh, Pennsylvania, USA; University of Pennsylvania, Philadelphia, Pennsylvania, USA

**Keywords:** enterovirus D68 (EV-D68), organoids, induced pluripotent stem cells, spinal cord organoids, acute flaccid myelitis (AFM), enterovirus, human model systems

## Abstract

Enterovirus D68 (EV-D68) is a nonpolio enterovirus associated with severe respiratory illness and acute flaccid myelitis (AFM), a polio-like illness causing paralysis in children. AFM outbreaks have been associated with increased circulation and genetic diversity of EV-D68 since 2014, although the virus was discovered in the 1960s. The mechanisms by which EV-D68 targets the central nervous system are unknown. Since enteroviruses are human pathogens that do not routinely infect other animal species, establishment of a human model of the central nervous system is essential for understanding pathogenesis. Here, we describe two human spinal cord organoid (hSCO)-based models for EV-D68 infection derived from induced, pluripotent stem cell (iPSC) lines. One hSCO model consists primarily of spinal motor neurons, while the another model comprises multiple neuronal cell lineages, including motor neurons, interneurons, and glial cells. These hSCOs can be productively infected with contemporary strains, but not a historic strain, of EV-D68 and produce extracellular virus for at least 2 weeks without appreciable cytopathic effect. By comparison, infection with hSCO with another enterovirus, echovirus 11, causes significant structural destruction and apoptosis. Together, these findings suggest that EV-D68 infection is not the sole mediator of neuronal cell death in the spinal cord in those with AFM and that secondary injury from the immune response likely contributes to pathogenesis.

**IMPORTANCE:**

AFM is a rare condition that causes significant morbidity in affected children, often contributing to life-long sequelae. It is unknown how EV-D68 causes paralysis in children, and effective therapeutic and preventative strategies are not available. Mice are not native hosts for EV-D68, and thus, existing mouse models use immunosuppressed or neonatal mice, mouse-adapted viruses, or intracranial inoculations. To complement existing models, we report two hSCO models for EV-D68 infection. These three-dimensional, multicellular models comprised human cells and include multiple neural lineages, including motor neurons, interneurons, and glial cells. These new hSCO models for EV-D68 infection will contribute to understanding how EV-D68 damages the human spinal cord, which could lead to new therapeutic and prophylactic strategies for this virus.

## INTRODUCTION

Enterovirus D68 (EV-D68) was isolated from patient respiratory samples in 1962 but is now considered an emerging pathogen due to increased circulation and implication in severe respiratory illness and acute flaccid myelitis (AFM), a polio-like illness causing paralysis, mostly in children (1 - 3). Large biennial outbreaks of EV-D68 and AFM were described from 2014 to 2018 (4). An additional outbreak of EV-D68 was expected in 2020; however, the transmission cycle was disrupted by nonpharmaceutical interventions used in the SARS-CoV-2 pandemic (5). EV-D68 returned in association with acute respiratory illness in the summer and autumn of 2022 with a high degree of circulation, but AFM rates nationally did not display an expected peak (6, 7). However, evidence supporting an association between EV-D68 and AFM has been mounting. Multiple reports have described enrichment of EV-specific antibodies in the central nervous system (CNS) of AFM patients, and EV-D68-positive staining was detected in the spinal cord of an autopsy specimen from a fatal case of AFM (8 - 10).

We previously reported that historic (prior to AFM) and contemporary (since AFM association) EV-D68 isolates differ in temperature- and acid-sensitivity as well as cellular tropism between respiratory and gastric epithelia, indicating virologic differences between contemporary and historic strains (11). However, mechanisms by which EV-D68 targets the CNS are unknown. This gap in knowledge is due in part to limitations in existing model systems. Enteroviruses are human pathogens and existing mouse models use neonatal or immunosuppressed mice or mouse-adapted viruses to study the relationship between EV-D68 and AFM (12, 13). Human spinal cord tissue is infrequently available for study, as patients rarely expire during their acute illness, and the spinal cord cannot be safely biopsied. Prior human CNS models for EV-D68 infection include neuronal cell lines and induced pluripotent stem cell (iPSC)-derived neurons or astrocytes (14 - 18) as well as brain organoids (19). However, EV-D68 is not a pathogen that commonly causes encephalitis.

To build on prior human models of CNS infection, we developed multicellular human spinal cord organoid (hSCO) models for EV-D68 infection. Based on published protocols for the cultivation of human spinal tissues from iPSC, we developed two hSCO models of EV-D68 infection (20, 21). The first is composed primarily of spinal motor neurons (SMNs), while the second contains multiple neural lineages. Embryologically, during the patterning of the neural tube, over 20 distinct classes of neurons are formed, orchestrated by the dorsal roof plate and the ventral floor plate (22). Using patterning morphogens that mimic the signaling from the roof and floor plates, a multicellular model can be generated that better represents the complexity of the human spinal cord functional unit. This model allows for development of multiple cell lineages of the spinal cord, including motor neurons, sensory neurons, glial cells, and interneurons (20).

Using these two model systems in concert, we discovered that hSCOs can be infected by EV-D68 isolates obtained after 2014 from multiple clades but not by older strains of EV-D68. EV-D68 induces minimal cell death in this system, suggesting that spinal cord damage during AFM is less likely due to death of individual infected neurons and more likely mediated by the anti-EV-D68 immune response. Thus, this model will be useful in understanding EV-D68 pathogenesis in the CNS.

## RESULTS

### Induced pluripotent stem cells can be differentiated into human spinal cord organoids

**Fig 1:**
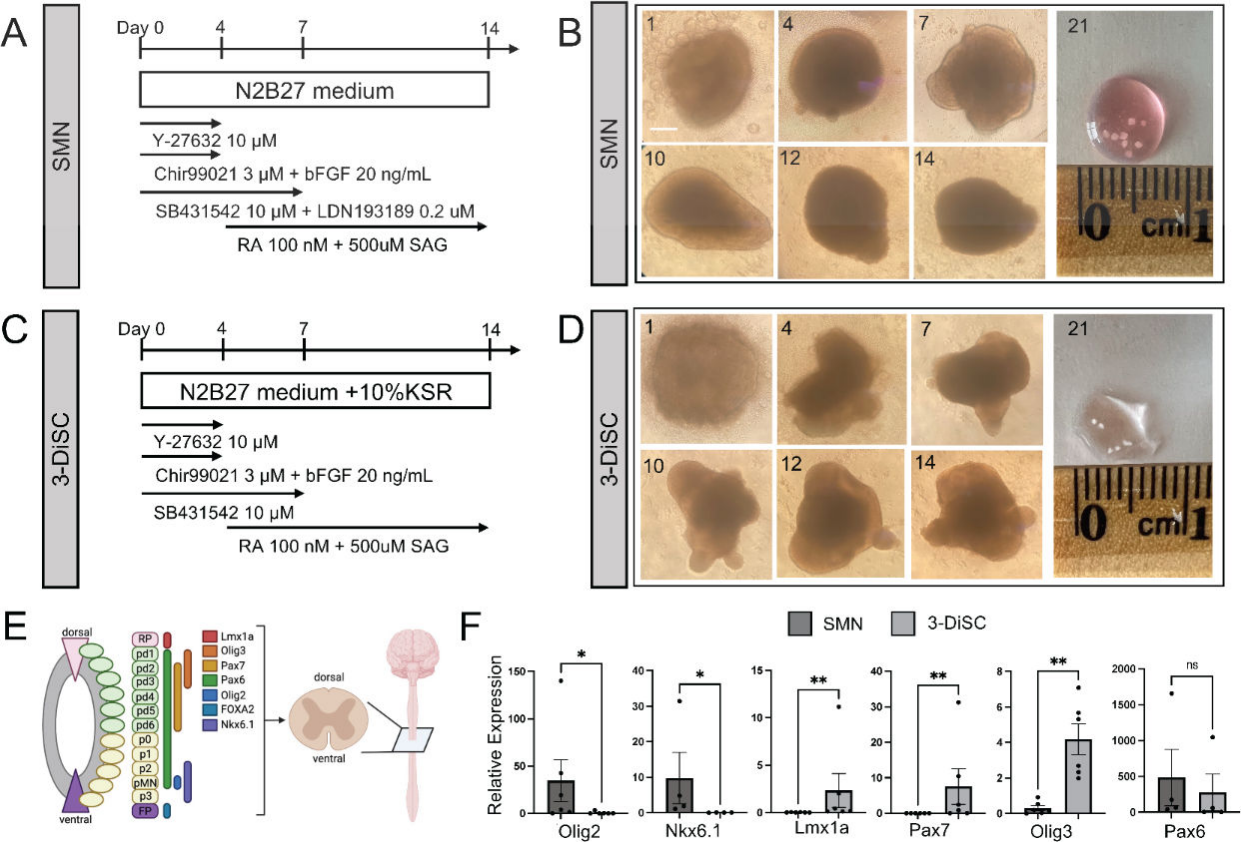
iPSCs can be differentiated into human spinal cord organoids. (**A and C**) Schematic showing differentiation protocol for spinal motor neuron (SMN) organoids (**A**) and 3-dimensional spinal cord (3-DiSC) organoids (**C**). (**B and D**) Morphologic evaluation of transition from iPSC aggregates to hSCO, SMN (**B**) and 3-DiSC (**D**). The day post-differentiation is marked in the upper left. (**E**) Schematic describing the expression pattern of progenitor markers in the spinal cord during development. Adapted from Ogura et al. (20). (**F**) qRT-PCR on day 14 comparing relative marker expression of SMN or 3-DiSC hSCO (*P < 0.05, **P < 0.01). Scale bar, 250 µm.

We prepared two different types of hSCO using established iPSC lines (20, 21). The first condition, SMN, comprises primarily spinal motor neurons. The second condition, 3-dimensional spinal cord (3-DiSC), has fewer applied growth factors, which allows development of increased cellular diversity along multiple differentiation pathways. Growth factors were applied for neural patterning for 2 weeks and varied by hSCO condition, as previously described (Fig. 1A through D) (20). hSCOs matured during this time, as evidenced by their cohesion and morphology detected using light microscopy (Fig. 1B and D). Additional growth factors (brain derived neurotrophic factor [BDNF], glial cell derived neurotrophic factor [GDNF], and retinoic acid) were applied to maintain and mature hSCOs past the differentiation protocol of the first 2 weeks. SMNs dominantly express Olig2 and Nkx6.1, ventral motor neuron markers (Fig. 1E through F), consistent with their spinal motor neuron composition. 3-DiSC hSCOs express multiple markers from along the neural tube (20), including LMx1a (roof plate/dorsal progenitor), Pax7 (dorsal progenitor), and Olig3 (dorsal progenitor) (Fig. 1E through F). While 3-DiSC hSCOs also express Olig2, expression is relatively less than that in SMN hSCO. Both SMN and 3-DiSC hSCO express Pax6, a marker present along most regions of the neural tube (Fig. 1E through F) (20, 21).

### EV-D68 infects human spinal cord organoids

**Fig 2:**
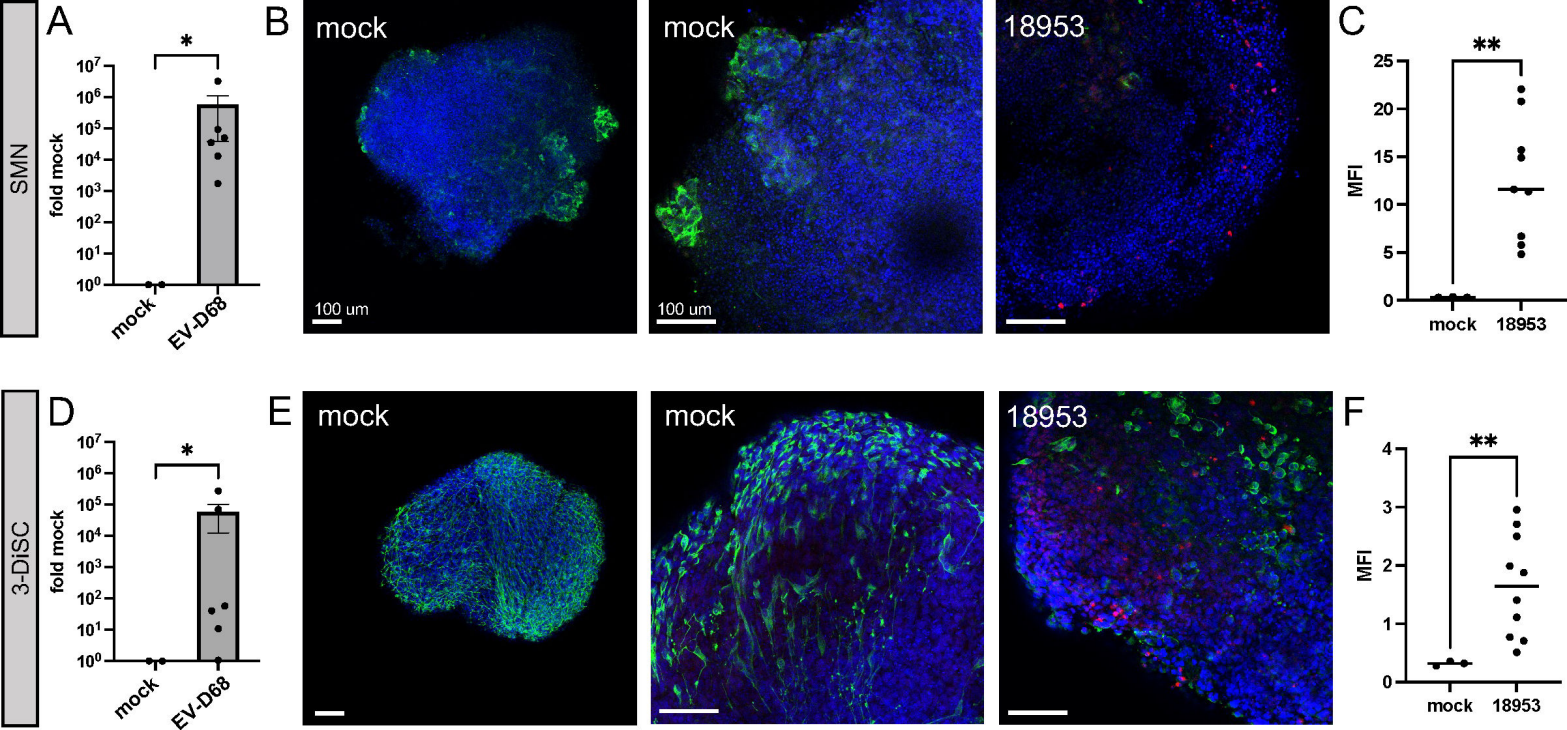
Contemporary EV-D68 infects human spinal cord organoids. (**A and D**) SMN (**A**) and 3-DiSC (**D**) organoids at 14 days post-differentiation in pools of 8–12 structures were either mock-infected or infected with 8.5 × 10^5^ PFU/well of US/KY/14-18953. At 48 h post infection (hpi), lysates were collected for qRT-PCR. (**B and C, E and F**) SMN (**B and C**) and 3-DiSC (**E and F**) hSCOs in pools of 8–12 were either mock-infected or infected with 10^5^ PFU/well US/KY/14-18953 and processed for immunofluorescence at 24 hpi. 4′,6-Diamidino-2-phenylindole (DAPI) in blue, neuron-specific beta III tubulin (tuj1) in green, and EV-D68 VP1 in red. Mean fluorescence intensity (MFI) for VP1 staining across multiple Z-stacks and individual organoids (*n* ≥ 3) is quantified for SMN (**C**) and 3-DiSC (**F**) (*P < 0.05, **P < 0.01). Scale bars, 100 µm.

**Fig 3:**
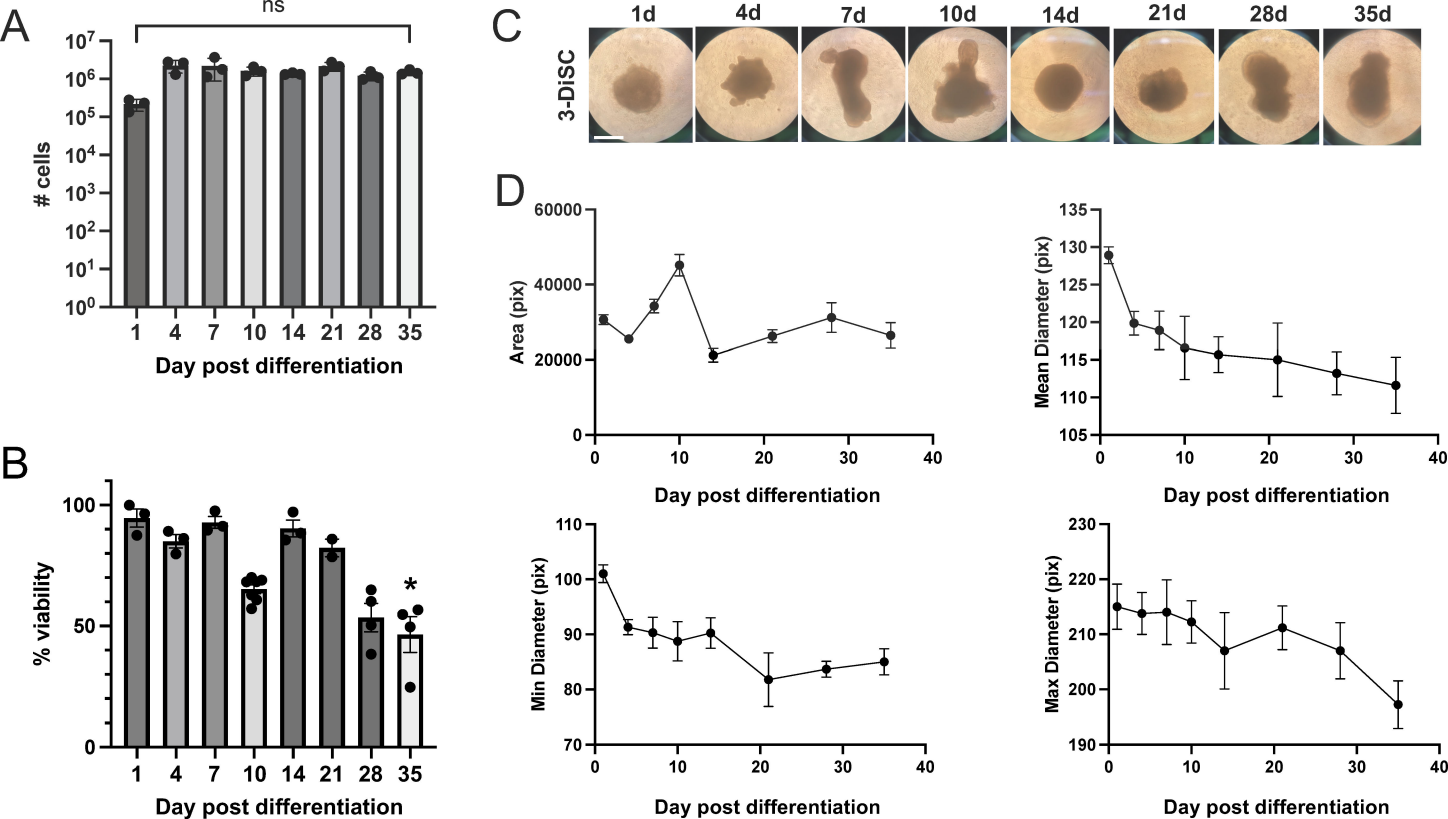
3-DiSC organoids maintain consistent cell number and viability. 3-DiSC hSCOs were differentiated per protocol and evaluated at multiple time points post-infection for the number of cells per structure (**A**), percent viability (**B**), and morphology (**C and D**). Structures were dissociated into single cells using Accumax and enumerated. Trypan blue staining was used to assess viability (*P < 0.05). Scale bar, 500 µm. Pix refers to pixels.

We tested the capacity of EV-D68 to infect SMN and 3-DiSC hSCO using US/KY/14-18953, a contemporary strain that circulated in 2014, an AFM peak year. After 14 days of differentiation, hSCOs were infected with US/KY/14-18953 and evaluated for expression of viral transcripts using qRT-PCR and for morphologic changes. Both SMN (Fig. 2A) and 3-DiSC hSCO (Fig. 2D) displayed abundant viral RNA 48 h post-infection (hpi) with US/KY/14-18953. For morphologic analysis, hSCOs at 14 days post-differentiation were either mock-infected or infected with US/KY/14-18953 for 24 h and then fixed and stained for neuron-specific beta III tubulin (tuj1) and EV-D68 VP1. The SMN condition (Fig. 2B and C) demonstrated a high degree of neural tubulin staining at regions of projection from the hSCO mass in both mock and infected hSCOs. For infected SMN hSCOs, VP1 was detected primarily at the hSCO periphery, but also was evident in Z-slices deeper into the interior of the organoid, as demonstrated by the range of VP1 MFI values throughout multiple fields of collected stacks (Fig. 2C). For 3-DiSC hSCO, neural tubulin had an even surface distribution for mock and infected hSCOs. Similar to observations made using SMN hSCO, VP1 was detected mostly at the periphery of the structures, but also was visualized deeper into the Z-stacks of the structure (Fig. 2E and F). Due to the increased cellular complexity of the 3-DiSC hSCO as well as equivalent infection susceptibility, we selected the 3-DiSC hSCO condition for additional studies.

### 3-DiSC organoids maintain cell number and viability

To define the optimal timing for viral infection of hSCOs, we investigated the cellularity, viability, and size of 3-DiSC hSCOs over time. hSCOs were differentiated per protocol and dissociated between days 1–35 post-differentiation. The number of cells per structure was comparable over time (Fig. 3A). We similarly dissociated 3-DiSC hSCOs and assessed cell viability using trypan blue staining. We found that cell viability was not significantly altered until 35 days post-differentiation, when viability dropped below 50% of quantified cells (Fig. 3B). Morphologically, the organoid structures evolved over time but maintained a relatively consistent area with age. Mean and minimum diameter initially decreased and then stabilized over time. Maximum diameter was relatively stable until a drop at the final timepoint at 35 days, which may have been related to increased cell death (Fig. 3C and D).

### EV-D68 infection of hSCO persists for at least 2 weeks

**Fig 4:**
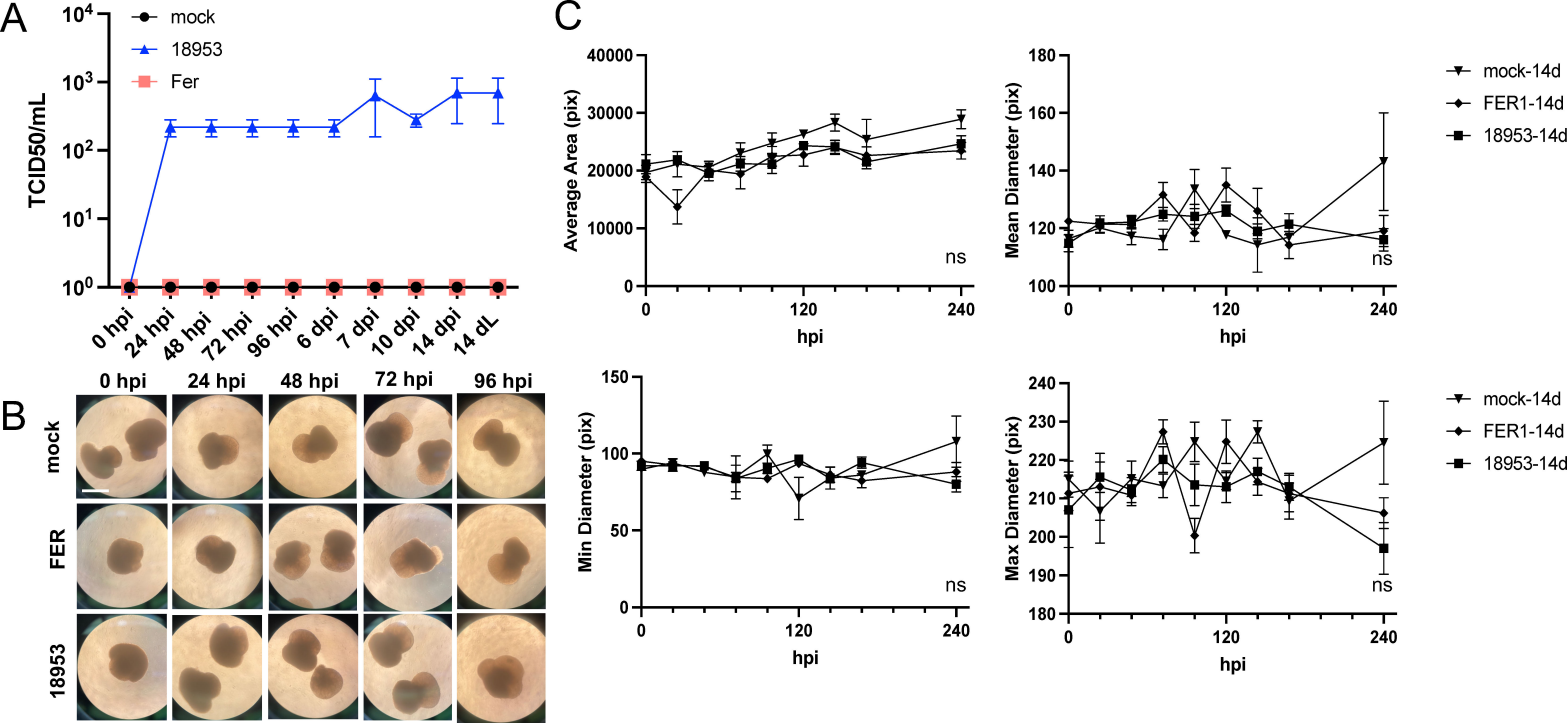
Productive EV-D68 infection does not alter hSCO morphology. 3-DiSC hSCOs 14 days post-differentiation were infected with 10^5^ PFU/mL of either Fermon or US/KY/14-18953 in pools of 12. Supernatants were collected for titer determination between 0 hpi and 14 days post infection (dpi). Terminal cellular lysate was collected at 14 days post infection (14 dL) (**A**). hSCOs were monitored daily for morphology for 10 days (**B and C**). Scale bar, 500 µm. Pix refers to pixels.

We tested the capacity of hSCO to model CNS infection with EV-D68 by initially comparing two EV-D68 strains. The Fermon strain was isolated in 1962 and is associated only with respiratory infection not AFM. Infection of 14-day post-differentiation hSCO with Fermon did not yield a productive infection, and no titer was detectable at any supernatant timepoint or in the terminal cellular lysate (14 dL) (Fig. 4A). We again used the US/KY/14-18953 strain as a contemporary neurotropic strain. US/KY/14-18953 caused a modest but sustained release of virus into the supernatant, with consistently detectable extracellular titer from 24 hpi through 2 weeks post-infection (Fig. 4A). When evaluated morphologically, hSCOs maintained their shape relative to mock-infected hSCOs during infection with either Fermon or US/KY/14-18953, without significant differences in mean area or mean, minimum, or maximum diameter among groups up to 10 days post-infection (Fig. 4B and C). Because 14-day-old hSCO structures are relatively nascent in terms of differentiation, we tested whether the kinetics of replication would be altered following further maturation of the organoids prior to infection. Similar results were obtained when hSCOs were aged to 21 days post-differentiation and infected with Fermon or US/KY/14-18953 (Fig. S1).

### Multiple clades of EV-D68 that emerged after 2014 productively infect hSCO

**Fig 5:**
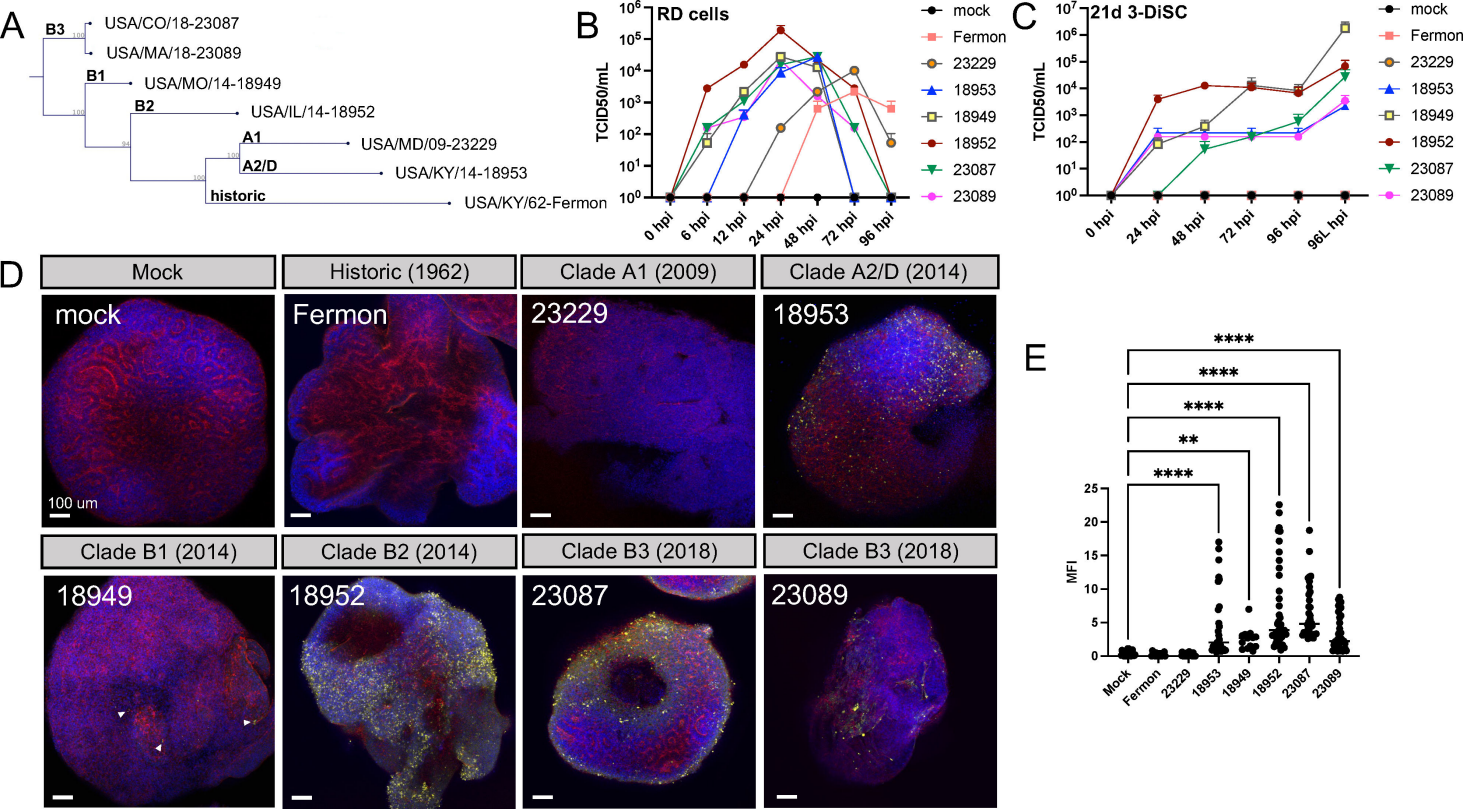
3D hSCOs can be infected with multiple contemporary strains of EV-D68. 3-DiSC hSCOs were infected with a panel of EV-D68 viruses as seen in the phylogenetic tree (**A**) representing multiple clades. These viruses all replicated robustly in RD cells (**B**), but more variably in 21-day post-differentiation 3-DiSC hSCO as demonstrated by titer (hpi) and terminal cellular lysate (96L hpi) (**C**). Fourteen-day post-differentiation 3-DiSC hSCOs in pools of 8–12 were either mock-infected or infected with 10^5^ PFU/well EV-D68 and processed for immunofluorescence at 24 hpi. DAPI in blue, actin in red, and EV-D68 VP1 in yellow. Arrowheads highlight VP1 signal (**D**). Mean fluorescence intensity (MFI) for VP1 staining is quantified for each strain across multiple Z-stacks and organoids (**N ≥ 3**) (**E**) (**P < 0.01, ****P < 0.0001). Scale bars, 100 µm.

Since EV-D68 has increased genetic diversity since 2014 and its association with AFM, we sought to evaluate infection of our hSCO model with strains representing each of the described EV-D68 clades, with the exception of Clade C, which has not circulated since 2010 (Fig. 5A). Each strain replicated efficiently in RD cells, which are susceptible to EV-D68 and often used for viral propagation (Fig. 5B). To evaluate viral replication in the hSCO model, we infected 21-day post-differentiation hSCOs with representative viral strains from all clades (Fig. 5C through E). As before, the Fermon strain did not produce measurable titer in replication assays or produce VP1 staining by immunofluorescence (Fig. 5C through E). Similarly, the Clade A1 isolate from 2009 (US/MD/09-23229) did not yield productive infection in the hSCO model (Fig. 5C through E). In contrast, infection with EV-D68 isolates from AFM years 2014 (Clade A2/D and Clade B1) and 2018 (Clade B3) productively infected hSCO, as evidenced by both supernatant and terminal cellular lysate (96L hpi) titers (Fig. 5C) and immunofluorescence staining (Fig. 5D and E) which is consistent with previously published neurotropism of EV-D68 strains from 2014 (12, 14, 15, 23, 24).

### EV-D68 infection with neurotropic strains is beyond the surface of the hSCO structure

**Fig 6:**
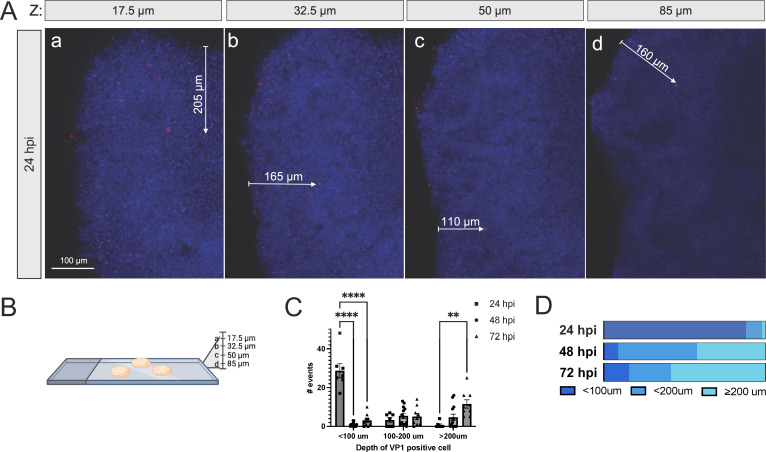
3-DiSC hSCOs are infected beyond the surface layer of cells. Pools of 8–12 3-DiSC hSCOs were infected for 24 h with US/MA/18/23089 and fixed and processed for confocal microscopy (**A**). Selections from the Z-stack are shown up to 85 µm in depth, as shown by the schematic (**B**). VP1 in red, with several infected cells identified with arrows measuring the distance from the structure surface. Scale bar, 100 µm. Pools of 8–12 3-DiSC hSCOs were infected for 24–72 h with US/MA/18/23089 and fixed and processed for confocal microscopy. The number of infected cells visualized was counted and grouped by distance from surface across multiple Z-stacks (17.5 µm or deeper into the stack) and individual organoids (**N3**). The data are shown as individual fields (**C**) and as total percentage over time (**D**) (**P < 0.01, ****P < 0.0001).

To determine whether EV-D68 can spread within the organoid cultures, 14-day post-differentiation hSCOs were infected with US/MA/18-23089 (B3 clade) for 24 h and processed for immunofluorescence microscopy (Fig. 6A and B). While much of the VP1 signal is evident at the surface level slice of the Z-stack or near the periphery of the organoid, infected cells can be identified greater than 200 µm into the interior of the structure, confirming that viral particles are not simply adherent to the exterior of the hSCO (Fig. 6A; Fig. S1). When infection was allowed to progress 48 or 72 h, cells that stained for VP1 were observed even further into the interior of the structure, supporting a role for trafficking deeper into the center of the organoid (Fig. 6C and D).

### EVD-68 infection of hSCO causes minimal apoptotic cell death

**Fig 7:**
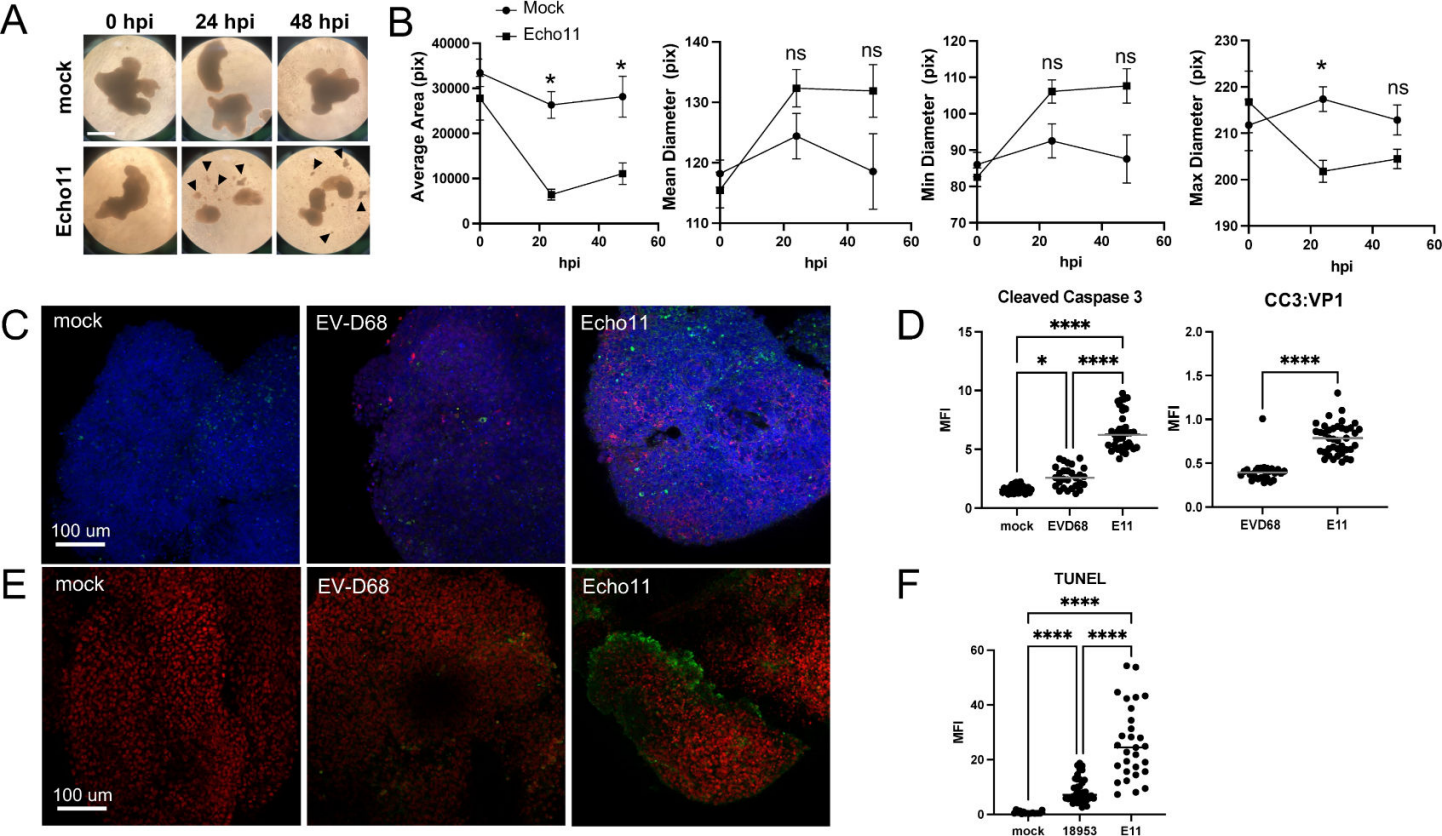
EVD-68 infection of hSCO causes less apoptosis than infection with E11. (**A and B**) 3-DiSC hSCOs at 14 days post-differentiation were infected with 10^5^ PFU/mL of echovirus 11 (**E11**). Morphology was evaluated at 24 and 48 hpi. Arrowheads mark cellular debris. (**C and D**) 3-DiSC hSCOs at 14 days post-differentiation were infected with 10^5^ PFU/mL of either US/KY/14-18953 or E11. hSCOs were fixed for immunofluorescence at 24 hpi and stained for VP1 (red), cleaved caspase 3 (green), and DAPI (blue). (**D**) MFI was quantified for cleaved caspase 3 (left) and normalized to VP1 signal (right) across multiple Z-stacks and individual organoids (**N ≥ 3**). (**E and F**) 3-DiSC hSCOs at 14 days post-differentiation were infected with 10^5^ PFU/mL of either US/KY/14-18953 or E11. hSCOs were fixed for immunofluorescence at 24 hpi and TUNEL assay was performed. Whole organoids were stained with anti-BrdU (green) to demonstrate DNA breaks and nuclei were stained with propidium iodide (PI). (**E**) MFI was quantified for TUNEL for each condition across multiple Z-stacks and individual organoids (**N ≥ 3**). (*P < 0.05, ****P < 0.0001). Scale bar, 100 µm.

As the architecture of hSCO was maintained during EV-D68 infection, we tested whether infection with EV-D68 caused cell death. As a control, we infected hSCO 14 days post-differentiation with echovirus 11 (E11). While not specifically a spinal cord pathogen, E11 uses the fetal Fc receptor (FcRN) for cell entry and causes neonatal meningoencephalitis (25). Therefore, we hypothesized that E11 would infect hSCOs. We observed that E11 produced a stark change in the morphology of the organoids, with disruption of the structures, decrease in size, and accumulation of cellular debris (Fig. 7A). When quantified, the average hSCO area and maximum diameter were decreased following infection with E11 (Fig. 7B). To assess for differences in cell death, we used quantitative immunofluorescence microscopy to detect viral protein expression and cleaved caspase 3, which is the active form of the protein during apoptosis (26, 27), in hSCO infected with either EV-D68 US/KY/14-18953 or E11 for 24 h. While infected hSCOs had abundant staining for EV-D68 and E11 VP1, infection with E11 induced significantly more cleaved caspase 3 than did infection with EV-D68 (Fig. 7C and D). This significant difference was maintained when signal was normalized to VP1 for each virus (Fig. 7D). To assess for differences in the late stages of the apoptosis pathway during these viral infections, we employed a terminal deoxynucleotidyl transferase (TdT) dUTP nick-end labeling (TUNEL) assay (28). TdT labels the blunt ends of double-stranded DNA breaks with incorporation of BrdUTP, which can be measured using immunofluorescence. We infected 14-day hSCOs with EV-D68 US/KY/14-18953 or E11 for 24 h and evaluated the cells for apoptosis using the TUNEL assay. Similar to our results with cleaved caspase 3, EV-D68 induced significantly more apoptosis than the mock-infected hSCO but significantly less than that induced by E11 infection (Fig. 7E and F).

## DISCUSSION

Development of 3D culture models began in the 1960s, although they became more prevalent in 2003, which paved the way for organoid models (29). The first stem-cell-based organoid model, an intestinal organoid, was published in 2009 (30). As stem cell biology has progressed, human iPSCs, first isolated in 2007, can be differentiated into organoids of multiple tissue types, without reliance on fetal tissues as a source of human stem cells (31). CNS models, first with cerebroids in 2013, and later spinal cord organoids in 2018, are a more recent development (32, 33). These models have mostly been used for studies of neurodevelopment or neurodegenerative conditions such as spinal muscular atrophy or amyotrophic lateral sclerosis (34, 35). Human organoid models of the spinal cord and brain are valuable tools to study human pathogens of the CNS in a multicellular system, in particular for pathogens that do not readily infect small animal models, as previously demonstrated for Zika virus (36, 37). We have established a model of EV-D68 infection of hSCOs using two previously established protocols for propagation of hSCOs from iPSC (20, 21).

EV-D68 is a nonpolio enterovirus that has been associated with severe respiratory disease and AFM in children since 2014, although the virus was originally discovered in 1962 (1). AFM is primarily a disease of children, with the average age of onset of 5 y (38). While AFM is a rare complication of EV-D68 infection, it causes serious morbidity in affected persons, often leading to lifelong medical needs. It is still unknown how EV-D68 targets the CNS to cause disease. Since humans are the only natural hosts for enterovirus infections, humanized models are required to study infection of this critical site. In this report, we show that hSCOs provide a relevant, multicellular model system for studies of contemporary EV-D68 strains. A contemporary EV-D68 isolate, but not the historic Fermon strain, infects both SMN and 3-DiSC hSCOs. This finding is consistent with prior studies suggesting that spinal motor neurons are the primary cell type infected by EV-D68 in the CNS, as that cell type is present in both hSCO models (12, 14, 15). This finding also recapitulates earlier studies showing that the Fermon strain does not infect neuronal cell lines (SH-SY5Y) or cause paralysis in mice (12, 14) but differs from another study that demonstrated that Fermon infects human iPSC-derived cortical neuron and astrocyte cultures (16). It is unknown whether these differences are attributable to differences in cell type and receptor expression, viral multiplicity of infection (MOI), or availability of exosomes, as recent studies have suggested that EV-D68 may use exosomal association as a receptor-independent cell-entry mechanism (17, 18).

Results gathered using our experimental organoid system are consistent with prior findings of CNS tropism *in vitro* and *in vivo* for Clades B1, B2, and A2/D EV-D68 strains, demonstrating the utility of hSCO for screening strains for neurotropism. Additionally, we have demonstrated that multiple 2018 isolates from the B3 clade also share the capacity to infect these hSCOs. Given that all available sequenced isolates from the 2022 season are also in the B3 clade, this model should be useful in probing the potential neurotropism of these strains, which will be particularly valuable, as there was not an associated peak of AFM cases in the 2022 season.

The precise transition between “historic/pre-AFM” and “contemporary/post-AFM strains” is not clear. While the Fermon strain from 1962 is definitively considered “historic,” as it was not associated with paralysis when it emerged (and coincided with a decrease in poliomyelitis presentations due to available vaccinations), there are few isolates available from the period prior to the formal tracking of AFM by the CDC in 2014. However, a case report identified EV-D68 as the likely cause of AFM in a patient from 2008 (10). This isolate is unfortunately unavailable for sequencing or laboratory study. An isolate in our studies from 2009, US/MD/09-23220, is not neurotropic in our hSCO model. Another strain of interest is the 2014 B1 strain 18949. While it productively infects hSCO (robust titer, especially in the cell lysate, but comparatively modest immunofluorescence signal) and replicates in SH-SY5Y cells, a neuronal cell line, it does not cause paralysis in mice (14, 39).

While productive infection of hSCO with contemporary EV-D68 was detected using several methods, including immunofluorescence, qRT-PCR, and extracellular titer, the overall quantified titer varied significantly by strain, and supernatant titer was uniformly less than the terminal cellular lysate titer. The hSCO model allowed continued infection with EV-D68 without excessive cell death or noted cytopathic effect (CPE), with titer detectable at least 2 weeks following infection. Similar observations have been reported when transitioning from a cell line model to primary cell cultures with RSV, in which infected primary airway cells produce a modest but detectable titer increase for 30 dpi without appreciable CPE (40, 41). Additionally, replication kinetics, cellular responses to infection, and cell-entry mechanisms may differ in neuronal and non-neuronal tissues (17, 18). Studies of poliovirus have demonstrated both delayed replication kinetics and decreased extracellular infectious virus in primary neuron cultures relative to fibroblast cells (42).

It is not known whether direct infection of motor neurons with EV-D68 or resultant injury to the spinal cord due to a pathogenic immune response is the primary mediator of paralysis in those with AFM. Previous studies using mice show loss of spinal motor neurons after infection (12). However, *in vitro* models of neuronal infection, including iPSC motor neuron cultures infected with EV-D68 (15) and primary neuron cultures infected with poliovirus (42), have not demonstrated CPE after infection. This observation could be due to alterations in receptor expression or cell-death machinery. In our model, EV-D68 US/KY/14-18953 infection induced apoptosis, as assessed by cleaved caspase 3 and TUNEL staining relative to mock-infected hSCO. However, EV-D68 infection was associated with significantly less apoptosis than was E11. Organoid morphology was maintained during EV-D68 infection for at least 2 weeks, suggesting that cell lysis induced by EV-D68 is relatively modest. Our experiments with E11 demonstrate that the hSCO model is capable of apoptosis when appropriately induced. For both viral infections, cells that stained for cleaved caspase 3 were not usually positive for viral antigen, suggesting that the apoptosis pathway is likely mediated by secreted factors such as cytokines. It also is likely that the absence of migratory components of innate and adaptive immunity in *in vitro* models allows EV-D68 infected cells to persist without the CPE seen *in vivo*. Previous studies with poliovirus have demonstrated that infection triggers apoptosis. However, productive infection also suppresses apoptosis, perhaps at a downstream stage (43). As for EV-D68, US/KY/14-18953 activates caspase 3 in RD cells, but there were no experiments conducted using single cells to test whether infected cells also stain for caspase 3 activation. Interestingly, inhibitors of caspase-3 decreased viral replication, and activators of caspase-3 increased viral replication, suggesting that this pathway may augment viral replication. Similarly, Fermon infection is associated with positive TUNEL staining in RD cells, but the cells were not co-labeled for viral antigen to test whether the DNA breaks occurred in infected or neighboring cells (44).

Although this multicellular model is an advanced and complementary to other existing models, it does have limitations. While the 3-DiSC condition does have many cell types, it has fewer than those that exist in the mature human spinal cord, which contains over 20 types of neurons that act in concert to form a cohesive tissue. Protocols exist to pattern 3-DiSC hSCO to become more cranial, caudal, dorsal, or ventral, but only one condition can be used at a time. Because growth factors are applied in a single well of a dish, the gradient provided by signals from the roof and floor plates is not present. Cross-field advances in bioengineering and biomaterials will be required to further increase the complexity of this model system. Additionally, this model lacks migratory innate immune cells as well as adaptive immune cells. Therefore, co-culture systems or complementary mouse experiments will be required to add the complexity of the immune response. Because this model differentiates only a few weeks prior to infection, the types of cells present are likely more immature than those infected in humans. However, despite these limitations, this human spinal cord model for EV-D68 will allow future in-depth studies of EV-D68 pathogenesis in the CNS, including studies of neurotropism of circulating viral strains, mechanisms of cell death, cellular response to infection, CNS-specific entry receptors, and testing antivirals in a relevant human *in vitro* model system.

## MATERIALS AND METHODS

### Cells and viruses

Human iPSCs (ACS-1030) were maintained in Pluripotent stem cell medium SFM SF/FF (ACS-3002) in flasks coated with 150 µg/mL Cultrex (R&D Systems, 3434-005-02). These cells were obtained commercially through the ATCC. Hela 7b cells were maintained in MEM medium supplemented to contain 5% FBS, 1% penicillin/streptomycin, and 1% NeAA. RD cells were maintained in DMEM medium supplemented to contain 10% FBS and 1% penicillin/streptomycin.

EV-D68 Fermon (ATCC VR-1826), US/KY/14-18953 (ATCC VR-1825), US/MD/09-23229 (CDC), US/MO/14-18949 (CDC), US/IL/14-18952 (ATCC VR-1824), US/CO/18/23087 (CDC), and US/CO/18-23089 (CDC) strains were propagated using Hela cells maintained at 33°C and 5% CO_2_ and purified using sucrose-cushion centrifugation as previously described (45). Echovirus 11 Gregory strain (ATCC VR-41) was propagated at 37°C and purified as above. Stocks were sequenced to ensure identity by VP1-specific primers as described previously (46).

### Propgation of human spinal cord organoid

iPSCs were dissociated to a single-cell suspension with Accumax and seeded at a density of 9,000 cells/well in 100 µL in a 96-well, round-bottom, low-adhesion plate as described (20). For SMN, hSCOs were plated in differentiation medium (N2B27 [DMEM/F-12 (Gibco, Billings, MT, USA), neurobasal medium (Gibco) (1:1), 0.5% (vol/vol) N2 supplement (ThermoFisher, Waltham, MA, USA) and 1% (vol/vol) B27 supplement without vitamin A (Invitrogen)] supplemented with 1 mM L-glutamine (L-Glu, Gibco), 0.1 mM 2-mercaptoethanol (2-ME, Sigma, St. Louis, MO, USA) and 0.5 µM ascorbic acid (Sigma). Every 3 days during differentiation, 50% of the medium was removed and replaced with fresh medium. For patterning, on days 0–3, 10 µM Y-27632 (Tocris, 1254), 20 ng/mL bFGF (ThermoFisher, 13256-029), and 3 µM CHIR 99021 were added. From day 0 to day 6, 10 µM SB431542 (Tocris, 1614) and 0.2 µM LDN 193189 (Tocris, 6053) were added. From day 3 to day 15, 100 nM retinoic acid (Tocris, 302-79-4) and 500 nM smoothened agonist (SAG) (Tocris, 912545-86-9) were added. For 3-DiSC hSCO, 10% (vol/vol) knockout serum replacement (KSR, Invitrogen) was added to differentiation medium, and LDN193189 and SAG were removed. After 14 days, organoids were transferred to a 24-well plate and cultured in suspension in N2B27 medium supplemented to contain 1 mM L-glutamine, 0.1 mM 2-ME, 0.5 µM ascorbic acid, 10 ng/mL BDNF (ThermoFisher, PHC7074), 10 ng/mL GDNF (ThermoFisher, PHC7041), and 100 nM RA.

### hSCO assays, dissociation, and infections

Organoids were dissociated to single-cell solutions using Accumax (Sigma, A7089) at 37°C and enumerated after staining with trypan blue. hSCO infections were completed in pools of 8–12 structures per well and inoculated with virus at an inoculum of 10^5^–10^6^ PFU/pool at room temperature for 1 h prior to 3× washes with PBS and transfer of infected hSCO to new wells prior to incubation with fresh medium at 33°C (EV-D68) or 37°C (E11) for the duration of the experiment. Media changes were no longer performed once an experiment began.^19^

### qRT-PCR, plaque assays, and TCID50s

Total RNA was isolated from hSCO using Sigma GenElute Total Mammalian RNA Miniprep Kit (Sigma, RTN350) and the Sigma DNase digest reagent (Sigma DNASE70). RNA (1 µg total) was reverse transcribed via iScript cDNA Synthesis Kit (Bio-Rad, Hercules, CA, USA, 1708891), diluted to 100 µL in ddH20, and analyzed using qPCR. RT-qPCR was conducted using iTaq Universal SYBR Green Supermix (Bio-Rad, 1725121) with a QuantStudio5 Instrument (Applied Biosystems, Waltham, MA, USA). Gene expression was quantified with the Δ*C*_Q_ method and normalized to human actin. Primer sequences are shown in Table 1. Plaque assays were conducted using Hela cells overlayed with 1% agarose, incubated at 33°C for 72 h (EV-D68) or 37°C for 48 h (E11) and 5% CO_2_ and stained with crystal violet prior to enumeration. TCID_50_ assays were conducted in triplicate and incubated at 33°C for 96 h (EV-D68) or 37°C (E11) and 5% CO_2_ and stained with crystal violet prior to enumeration.

**TABLE 1:** Table of primer sequences

Target	Forward	Reverse
EVD68	5′-TGTTCCCACGGTTGAAAACAA	5′-TGTCTAGCGTCTCATGGTTTTCAC
h_actin	5′-ACTGGGACGACATGGAGAAAAA	5’- GCCACACGCAGCTC
h_olig2	5′-GGGCCACAAGTTAGTTGGAA	5′-GAGGAACGGCCACAGTTCTA
h_olig3	5′-CCTGCTCGCCAGAAACTACA	5′-CCCCATAGATCTCGCCAACC
h_nkx6.1	5′-CACACGAGACCCACTTTTTCC	5′-CCCAACGAATAGGCCAAACG
h_lmx1a	5′-TCAGAAGGGTGATGAGTTTGTCC	5′-GGGGCGCTTATGGTCCTTG
h_pax6	5′-TCTTTGCTTGGGAAATCCG	5′-CTGCCCGTTCAACATCCTTAG
h_pax7	5′-CTTCAGTGGGAGGTCAGGTT	5′-CAAACACAGCATCGACGG

### Immunofluorescence (IF) and fructose-glycerol clearing

IF labeling was conducted using the fructose-glycerol clearing method as described (47). hSCOs were fixed in 4% PFA and washed in 1% PBS-BSA and PBS-T at 4°C. Remaining washes used organoid wash buffer (OWB, 0.1% triton-X-100 (v/v), 0.2% BSA (m/v) in PBS). Primary antibodies were used at 1:250 and included VP1 (Genetex, Irvine, CA, USA, GTX132313), Tuj-1 (ThermoFisher, 480011), NCL-ENTERO (clone 5-D8/1, Leica Biosystems, Wetzlar, Germany), and cleaved caspase 3 (Asp 175) (Cell Signaling Technology, Danvers, MA, USA, 9661). Secondary antibodies were used at 1:1000. hSCOs were cleared with 60% glycerol in 2.5M fructose for 30 min prior to mounting and imaging using a Leica Stellaris 5 confocal microscope. Images were processed using FIJI 2 version 2.9.0.

### TUNEL assay

The APO-BrdU TUNEL Assay (Invitrogen Cat. A23210) was used according to the manufacturer’s instructions and applied to whole hSCO. Incubation times were modified to adjust for tissue size. hSCOs were incubated in 70% EtOH for at least 24 h prior to additional processing. The DNA-labeling step was allowed to proceed for 24 h at 37°C. Antibody labeling steps were conducted using the IF strategies described above.

### Statistical analysis

Statistical analysis was conducted using GraphPad Prism software version 9.4.1. All data are shown as mean ± SEM. Experiments were conducted in biologic triplicate. One-way ANOVA and Student’s *t*-tests were used to determine significance, as appropriate. When data had a normal distribution, per D’agostino-Pearson analysis, parametric tests were applied. Otherwise, nonparametric tests were used. *P* values are noted in figure legends, with *P* < 0.05 considered significant.

## SUPPLEMENTAL MATERIAL

Figure S1 mbio.01058-23-s0001.pdf Productive EV-D68 infection of more mature hSCOs does not alter hSCO morphology.

Supplemental Legends mbio.01058-23-s0002.docx Legends for Fig. S1 and Video S1.

Video S1 mbio.01058-23-s0003.avi EV-D68 infected cells are present beyond hSCO periphery. Z-stack reconstruction of a 14-day post-differentiation 3-DiSC hSCO infected with US/MA18/23089. Nuclei-DAPI, blue, EV-D68 VP1 in yellow.

ASM does not own the copyrights to Supplemental Material that may be linked to, or accessed through, an article. The authors have granted ASM a non-exclusive, world-wide license to publish the Supplemental Material files. Please contact the corresponding author directly for reuse.
