# Supplementary figures and images for: Contemporary enterovirus-D68 isolates infect human spinal cord organoids
Source: mBio. 2023 Aug 3;14(4):e01058-23. doi: 10.1128/mbio.01058-23 (PMC10470749; doi:10.1128/mbio.01058-23)

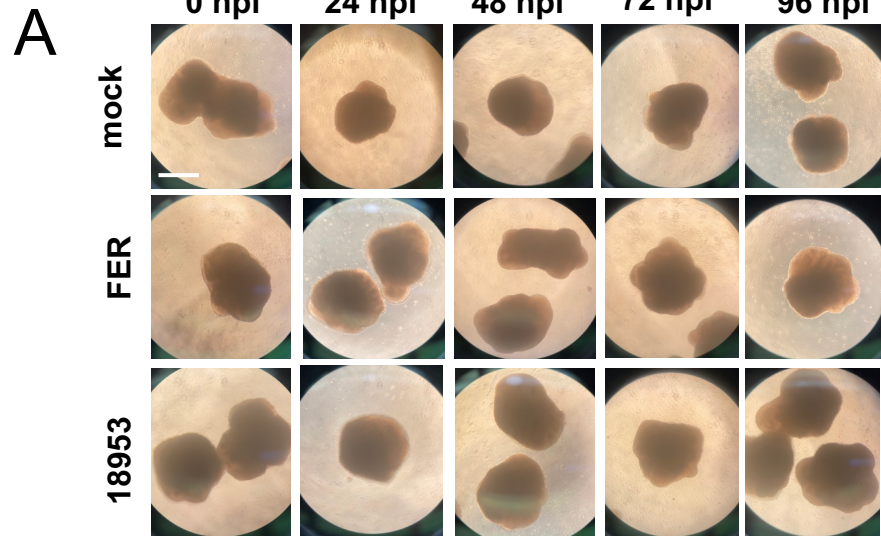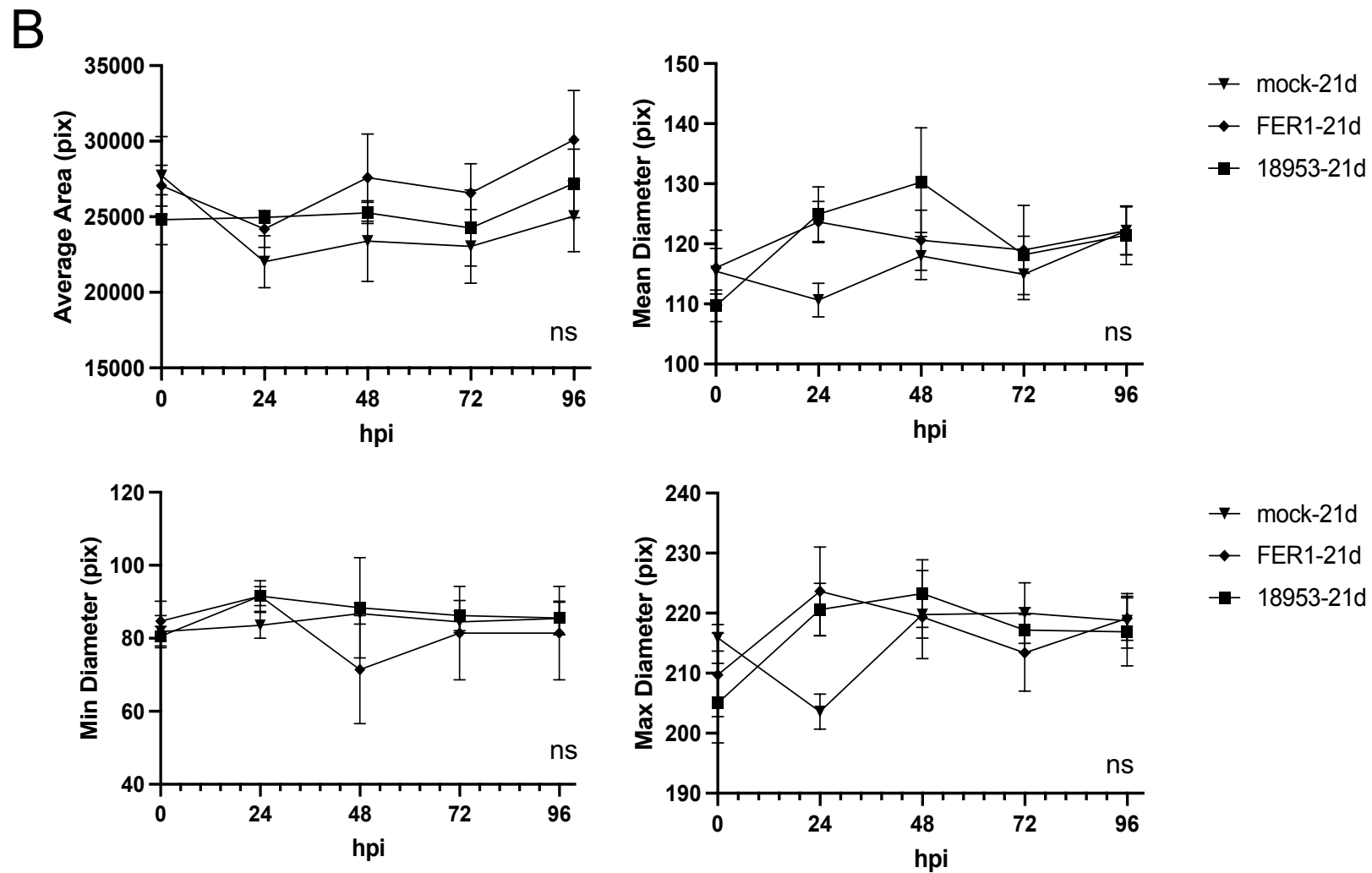

Supplement: Figure S1 — Productive EV-D68 infection of more mature hSCOs does not alter hSCO morphology. [file mbio.01058-23-s0001.pdf]
