# Supplementary material for: Contemporary enterovirus-D68 isolates infect human spinal cord organoids
Source: mBio. 2023 Aug 3;14(4):e01058-23. doi: 10.1128/mbio.01058-23 (PMC10470749; doi:10.1128/mbio.01058-23)
Supplement: Supplemental Legends — Legends for Fig. S1 and Video S1. [file mbio.01058-23-s0002.docx]

**FIG S1** Productive EV-D68 infection of more mature hSCO does not alter hSCO morphology. 3-DiSC hSCO 21 d post-differentiation were infected with 10^5^ PFU/mL of either Fermon or US/KY/14-18953 in pools of 12. hSCO were monitored daily for morphology for four days (**A-B**). Scale bar, 500 µm. Pix refers to pixels.

**FIG Video 1** EV-D68 infected cells are present beyond hSCO periphery.

Z-stack reconstruction of a 14-d post differentiation 3-DiSC hSCO infected with US/MA18/23089. Nuclei-DAPI, blue, EV-D68 VP1 in yellow.
